# A Commentary on “Prognostic significance of the albumin-to-alkaline phosphatase ratio in gastrointestinal cancer patients: a systematic review and meta-analysis”

**DOI:** 10.1097/JS9.0000000000003280

**Published:** 2025-08-25

**Authors:** Yahui Sun, Miaomiao Ren, Xuyang Sun

**Affiliations:** a Department of Gastrointestinal Surgery, Hainan General Hospital (Hainan Affiliated Hospital of Hainan Medical University), Haikou, Hainan Province, China; b Operating Room, Hainan General Hospital (Hainan Affiliated Hospital of Hainan Medical University), Haikou, Hainan Province, China; c Department of Interventional Radiology, Hainan Hospital of the General Hospital of the People’s Liberation Army of China, Sanya, Hainan Province, China

*Dear Editor,*

We read with great interest the study conducted by Zhu *et al*^[1]^, which investigated the prognostic value of the albumin-to-alkaline phosphatase ratio (AAPR) in gastrointestinal (GI) cancer patients. This meta-analysis, encompassing eight studies involving 2267 patients, revealed that AAPR is an independent risk factor for overall survival, but not for recurrence-free survival. Nevertheless, several aspects require further elucidation prior to translating these findings into clinical practice.

First, we cannot concur with the author’s definition of the research population. In this particular study, GI cancer was defined as encompassing only gastric cancer and colorectal cancer, which clearly deviates from the established definition of GI cancer. Herein, we reassert the definition of GI cancer as follows: GI cancer pertains to malignant conditions of the GI tract and accessory digestive organs, including the esophagus, stomach, biliary system, pancreas, small intestine, large intestine, rectum, and anus^[2]^.

Secondly, a comprehensive literature search is an indispensable prerequisite for ensuring the reliability of a meta-analysis. Based on the search strategy presented by the author, we discovered that the author overlooked several crucial studies. Regarding esophageal cancer, Zhu *et al*^[3]^ revealed that a lower AAPR was a significant prognostic factor leading to poor overall survival and progression-free survival in patients who underwent esophagectomy. In the case of hepatocellular carcinoma, eight studies reported in a previously published meta-analysis^[4]^ also satisfied the inclusion criteria. Therefore, it would have been advisable for the authors to incorporate these studies into their analysis to more accurately assess the prognostic value of the AAPR in GI cancers.

Thirdly, relying solely on small *I*^2^ values to employ a fixed-effects model for pooled outcomes is inappropriate^[5]^. Among the research characteristics of the included studies, substantial differences were observed in tumor types, initial treatment modalities, cutoff values of AAPR, and the sampling time of serum samples. It is highly probable that the included patients exhibit heterogeneity. Consequently, applying a random-effects model across the pooled analysis is more likely to be suitable in such a scenario.

Overall, we express our sincere gratitude to Zhu and colleagues for their efforts in exploring the prognostic value of AAPR in patients with GI cancer. However, we would like to raise some concerns, which may potentially help the authors improve the clarity of their research findings.

This study is compliant with the TITAN Guidelines 2025^[6]^.

## Data Availability

No data used in this letter to the editor.

## Research registration unique identifying number (UIN)

No need for this letter to the editor.

## References

[1] Zhu XM Bai X Liu HZ Dai DQ. Prognostic significance of the albumin-to-alkaline phosphatase ratio in gastrointestinal cancer patients: a systematic review and meta-analysis. Int J Surg 2025. 10.1097/JS9.0000000000003032 PMC12626576 40717590

[2] Arnold M Abnet CC Neale RE. Global burden of 5 major types of gastrointestinal cancer. Gastroenterology 2020;159:335–349.e315. 32247694 10.1053/j.gastro.2020.02.068 PMC8630546

[3] Zhu X Chen D Li S. Albumin-to-alkaline phosphatase ratio as a novel and promising prognostic biomarker in patients undergoing esophagectomy for carcinoma: a propensity score matching study. Front Oncol 2021;11:764076. 34746006 10.3389/fonc.2021.764076 PMC8563791

[4] Ibn Awadh A Alanazi K Alkhenizan A. Prognosis of hepatocellular carcinoma using the albumin to alkaline phosphatase ratio, literature review, and meta-analysis. Ann Med Surg Lond 2024;86:6062–70. 39359833 10.1097/MS9.0000000000002375 PMC11444611

[5] Pang H Sun H. Considerations in meta-analysis of immunotherapy for advanced cancers other than melanoma. JAMA Oncol 2024;10:535–36. 38300567 10.1001/jamaoncol.2023.6770

[6] Agha RA Mathew G Rashid R. Transparency In The reporting of Artificial Intelligence – the TITAN guideline. Prem J Sci 2025;10:100082.

